# Parliament and the Profession

**Published:** 1917-12-22

**Authors:** 


### Parliament and the Profession.

### Enteric Fever in the Navy and Army.

DR. MACNAMARA informed Mr. Chancellor, who asked how many of the 893 cases of enteric fever referred to in the Local Government Board's Report for 1916-17 were naval, that there were forty cases in the Navy in the period referred to, one patient had been inoculated and survived, nine cases proved fatal. A similar question in respect of the Army elicited the reply from Mr. Macpherson that the information is not available.

### Dietaries of Workhouse Infirmary Patients.

MR. WALSH stated that the feeding of sick and infirm patients is the responsibility of the medical officers of institutions, and these persons are not subject to the Food Controller's scales imposed upon able-bodied inmates by the Local Government Board's directions. He had not heard of any instance of insufficient dietaries, but would cause inquiries to be made.

### Dentists and Service in a Volunteer Force.

MR. STEPHEN WALSH said that the President of the Local Government Board has received representations, in respect of Tribunals requiring exempted dentists to join a Volunteer Training Corps, from the Dental Service Committee. Such a condition, although not *ultra vires*, should not be imposed unless it is clear that the man can spare the time and that it is in the national interest. A condition of exemption should preferably take the form of requiring such additional dental service as may be recommended by the Dental Service Committee.

### Medical Prisoners of War

MAJOR HUNT asked the Under-Secretary of State for War why French medical officers captured by the Germans are released within about a week, whilst British medical officers are kept as prisoners for months. Mr. Macpherson said he would be glad to receive information on this point, not being aware that the French are better treated in this respect than the British.

### Voluntary Hospitals and Grants for Soldiers.

BEING asked by Sir William Collins whether he was in a position to state what increases in the scales of grants to voluntary hospitals, in respect of treatment of wounded soldiers, had been decided upon, Mr. Forster, Financial Secretary to the War Office, said that the maximum grant for Class A Auxiliary Hospitals had been raised from 3s. to 3s. 6d. per day, and for Class B from 2s. to 2s. 6d.

